# The Biochar Derived from Pecan Shells for the Removal of Congo Red: The Effects of Temperature and Heating Rate

**DOI:** 10.3390/molecules29235532

**Published:** 2024-11-22

**Authors:** Wanqiang Xu, Bo Cai, Xujie Zhang, Yating Zhang, Yongjian Zhang, Hehuan Peng

**Affiliations:** 1 College of Optical, Mechanical and Electrical Engineering, Zhejiang A & F University, Hangzhou 311300, China; 2 College of Chemistry and Materials Engineering, Zhejiang A & F University, Hangzhou 311300, China; 3 Key Laboratory of Agricultural Equipment for Hilly and Mountainous Areas in Southeastern China (Co-Construction by Ministry and Province), Ministry of Agriculture and Rural Affairs, Hangzhou 311300, China

**Keywords:** pecan shell, pyrolysis, biochar, Congo Red, adsorption

## Abstract

Organic pollutants, especially dyes, are seriously hazardous to the aquatic system and humans due to their toxicity, and carcinogenic or mutagenic properties. In this study, a biochar prepared from agricultural waste (pecan shells) via pyrolysis was applied to remove the dye pollutant Congo Red from wastewater to avoid a negative effect to the ecosystem. This study also investigated the effect of preparation conditions (temperature and heating rate) on the physicochemical properties and the adsorption performance of biochars. The physicochemical properties of the biochar were characterized using scanning electron microscopy, X-ray powder diffraction, Fourier transform infrared spectroscopy, and X-ray photoelectron spectroscopy. The adsorption performance of the biochar was evaluated for Congo Red removal. The results showed that biochar prepared at 800 °C with a heating rate of 20 °C/min (PSC-800-20) exhibited a higher specific surface area of 450.23 m^2^/g and a higher adsorption capacity for Congo Red (130.48 mg/g). Furthermore, adsorption experiments indicated that the pseudo-second-order and Langmuir models fitted well with the adsorption kinetics and isotherms of the biochar derived from pecan shells, respectively. Additionally, the PSC-800-20 biochar demonstrated a stable adsorption capacity over multiple cycles, suggesting its potential for regeneration and reuse in wastewater treatment applications. Therefore, the biochar derived from agricultural waste presents a promising and sustainable solution for the removal of toxic dye pollutants from wastewater.

## 1. Introduction

Organic dyes have been widely applied in the textile, paper, leather industries, and other related industries [1]. However, the discharge of dye wastewater into the aqueous ecosystem interrupts reoxygenation and inhibits the photosynthesis of aquatic organisms, seriously affecting their normal life activities [2]. Furthermore, some dyes with high toxicity and carcinogenic or mutagenic properties would be harmful to human’s health and the ecological environment [3]. Among these dyes, Congo Red (CR), an anionic dye, is physiologically toxic (such as in terms of cell damage, oxidative stress, and gene mutations) for the environment and humans, which not only disrupts the normal functioning of aquatic ecosystems but also inhibits the growth of algae and phytoplankton, thus reducing the number of primary producers in the water, which in turn impacts the entire food chain [4,5,6,7]. Hence, dye wastewater should be treated before discharging. Several treatment techniques have been developed for the removal of the dye from wastewater, such as biological degradation, photochemical degradation, coagulation, flotation, and adsorption [8,9,10]. Among these approaches, adsorption is considered to be the quickest and most effective way for dye wastewater treatment [11]. In recent years, various adsorbents (e.g., porous biochar, activated carbon, magnetic materials, and functional polymers) have been explored to improve efficiency for the highly efficient removal of dyes from wastewater [12].

Biochars are produced from forestry wastes, agricultural wastes, and sludge, etc. at certain temperatures and oxygen conditions [13]. Meanwhile, biochars possess a porous structure and physicochemical properties, including a large specific surface area and aromaticity [14]. Biochars derived from various biomass wastes (such as poplar, rick husk, rice, wheat straw) have been developed for wastewater treatment [15,16,17,18]. Li et al. reported the use of biochars derived from bamboo sawdust for the removal of Congo Red (CR) from aqueous solutions, with a maximum adsorption capacity of 33.7 mg g^−1^ [6]. In contrast, Alberto A. et al. prepared a magnetic nanocomposite (MNC) of γ-Fe_2_O_3_ nanoparticles (NPs) and polypyrrole (PPY) that was synthesized by the in situ chemical oxidative polymerization of pyrrole in the presence of FeCl_3_ as an oxidizing agent to evaluate its adsorption capacity of Congo Red (CR) in aqueous solutions. The maximum adsorption capacity (q_e_) achieved was 269.5 mg g^−1^ at a pH of 7.0 and 60 min of interaction time [19]. Global walnut production exceeds 3 million tons; pecan are an important agricultural product [20,21]. Unfortunately, a large number of pecan shells ensue, which are deemed to be an agricultural waste, and if not be utilized, cause serious environmental pollution owing to their difficult biodegradation [22]. Therefore, the resource utilization of pecan shells has become urgent. The preparation of the biochar from pecan shells not only effectively solves the issue of wasted resources, but biochars also serve as an eco-friendly and economical adsorbent for wastewater treatment. The biochar derived from pecan shells has been reported as an absorbent for the removal of hexavalent chromium from aqueous solutions, which exhibit an Cr (VI) adsorption capacity of 35.4 mg g^−1^ [23]. Furthermore, Komnitsas et al. prepared biochars derived from pecan shells over a temperature range of 250–550 °C, then evaluated their adsorption performance via the removal of Pd and Cu from the solutions [24]. The result indicated that temperature is a crucial factor for physicochemical properties and the adsorption capacity of biochars. In addition, many studies have suggested that pyrolysis temperature and heating rate presented significant effects on the structure of biochars, which would further affect the performance of biochars [25,26,27,28]. However, most of these studies have indicated that the adsorption performance was affected by the specific surface area and pore structure, while the influence of physical and chemical properties on adsorption performance should be further explored.

In this study, the biochar was fabricated from pecan shells at different temperatures and heating rates and then applied as the adsorbent for the removal of CR dye from wastewater. The physicochemical properties of biochars were investigated via various characterization techniques. Meanwhile, the adsorption process of CR was evaluated via the adsorption kinetics and adsorption isotherms. Based on the results of the characterization and experiments, the relationship between the physicochemical properties and the adsorption performance of biochar was investigated. The result in this study will show the effects of temperature and heating rate on the structure and adsorption performance of biochars, which could be the reference for the controlled preparation of biochar and improve the adsorption capacity of organic dyes in wastewater.

## 2. Results and Discussion

### 2.1. The Characterization of Biochar Derived from Pecan Shells

**Figure 1:**
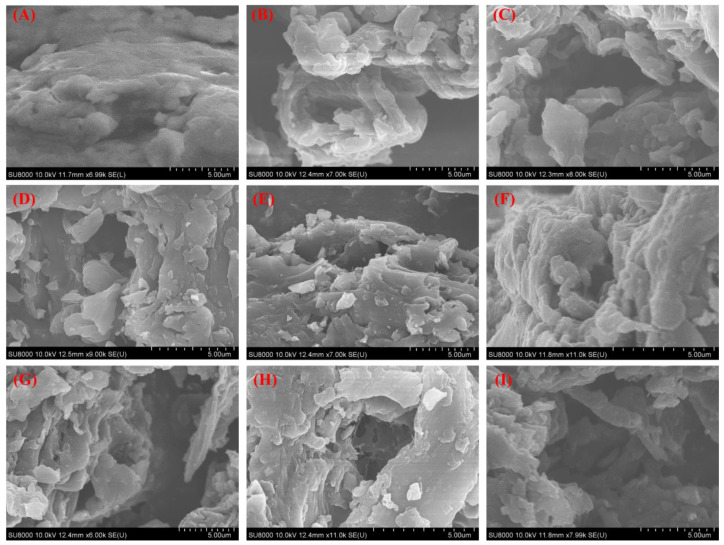
SEM images of biochar derived from pecan shell. (**A**–**I**) PS, PSC-400, PSC-500, PSC-600, PSC-700, PSC-800-5, PSC-800-10, PSC-800-20, PSC-900, respectively.

The morphology and element content of PSs and the biochars derived from PSs were examined by SEM, as shown in Figure 1 and Table S1. The PS feedstock presented a dense surface morphology surface, while loose morphology was obtained after the carbonization process. Furthermore, the SEM images of the biochars prepared at different temperatures are shown in Figure 1B–E,G,I. The surface morphology of the biochars gradually became looser as the carbonization temperature increased, which could be attributed to the degradation of the unstable components and the generation of a gaseous product at the high pyrolysis temperature, thereby leading to the formation of much more pores [29]. However, the PSC-900 displayed denser morphology than PSC-800, which might have been caused by the shrinkage and aggregation of the biochar at the high temperature. This phenomenon suggested that the morphology of the biochar was affected by the carbonization temperature. In addition, the influence of the heating rate on the surface morphology of the biochar was analyzed. Figure 1F–H shows the morphology of the biochar prepared with different heating rates (5 °C/min, 10 °C/min, 20 °C/min). More debris was observed in the biochar prepared at 20 °C/min, which may be due to the fast release of volatiles during the carbonization process. The analysis on the morphology of the biochar indicated that the physical characteristics could be tuned by controlling the parameters of the carbonization process. Meanwhile, the element composition of the PSC-800-10 biochar was determined by SEM-EDS (Figure S1), which showed that the biochar mainly consisted of carbon and oxygen, and as we know, hydrogen is also one of the components of biochars.

**Figure 2:**
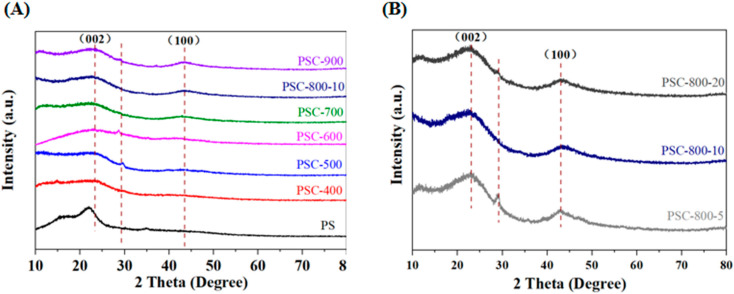
The X-ray diffraction spectra of biochar derived from pecan shell. (**A**) Biochar prepared at different temperatures; (**B**) biochar prepared at different heating rates.

The XRD spectra of the pecan shell and the as-obtained biochar are presented in Figure 2. The typical diffraction peaks of cellulose appeared (at 15.5°, 22.4°, and 34.4°) in the XRD spectra of the pecan shell, while the diffraction peaks of these could not be observed in the biochar sample, which indicated that the components of the pecan shell were converted into carbon during the calcination process (Figure 2A). Notably, the XRD spectra of the biochar presented two similar diffraction peaks, where the former broad peak at 23° was assigned to the (002) plane of the amorphous carbon and the latter peak at 43.3° was indexed to the (100) plane of the crystalline carbon [30]. Furthermore, the intensity of the diffraction peaks at 43.3° increased as the temperature increased, which showed that the high calcination temperature would be beneficial for the formation of a graphitic structure [31,32]. This result indicated that the crystalline structure of the biochar could be regulated by a change in temperature. In addition, the influence of the heating rate on the biochar structure was explored when the carbonization temperature was at 800 °C, as shown in Figure 2B. The border and lower diffraction peaks at 23° and 43.3° could be found in the XRD pattern of the biochar prepared at a higher heating rate, which indicated that the amorphous carbon could be easily formed at the high heating rate. This result could be attributed to the temperature-sensitive components (hemicellulose and cellulose) being converted into more gaseous products, which would not be conducive to the formation of the crystalline structures of biochars. In addition, a tiny diffraction peak at 29.1° could be found at the biochars prepared at a low temperature and heating rate (PSC-500, PSC-600 and PSC-800-5), which was attributed to the salt of the alkaline earth metals [33]. Meanwhile, the peak disappeared at a high temperature and heating rate, indicating that the high temperature and heating rate were favorable for the release of metals, which could further optimize the pore structure of the biochar. Overall, the calcination temperature and heating rate were important influencing factors on the structure of the biochar derived from the pecan shell.

The textural characterization of the biochar derived from the pecan shell was determined via N_2_ adsorption–desorption analysis, as shown in Figure S2 and Table 1. According to the IUPAC classification, Figure S2A,B shows that all the biochars displayed type-II curves, which suggests that the structure of biochars is microporous [34]. Also, the pore size distribution curves of the biochars are shown in Figure S2C,D. It can be observed that the PSC-800-20 exhibited a micro-mesoporous structure, while the other biochar exhibited a microporous structure. The micro-mesoporous structure of the PSC-800-20 would be beneficial for mass transfer during the adsorption process of dyes [35]. Additionally, Table 1 presents detailed information about the specific surface area and pore structure of biochars.

The textual properties of the biochars were summarized in Table 1. The pecan shell was pyrolyzed at 800 °C with 10 °C/min; the specific surface area of the biochar reached the maximum value (436.4 m^2^ g^−1^). When the temperature further increased to 900 °C, the specific surface area of the biochar decreased, which may be due to the collapse of the tiny pores. It can be observed that the specific surface area and pore volume of the biochar increased as the temperature increased when the heating rate remained at 10 °C/min, which suggests that the pyrolysis temperature was an important impact on the biochar structure. Then, the pyrolysis temperature remained at 800 °C so that we could explore the influence of the heating rate on the biochar structure. The biochar prepared with a high heating rate exhibited a higher specific surface area and pore volume. In addition, the Figure S2D showed some pore sizes at 2–4 nm in the PSC-800-20, which indicated that the PSC-800-20 possessed some mesopores in the pore structure. Overall, the temperature and heating rate affected the specific surface area and porous structure of the biochar.

**Figure 3:**
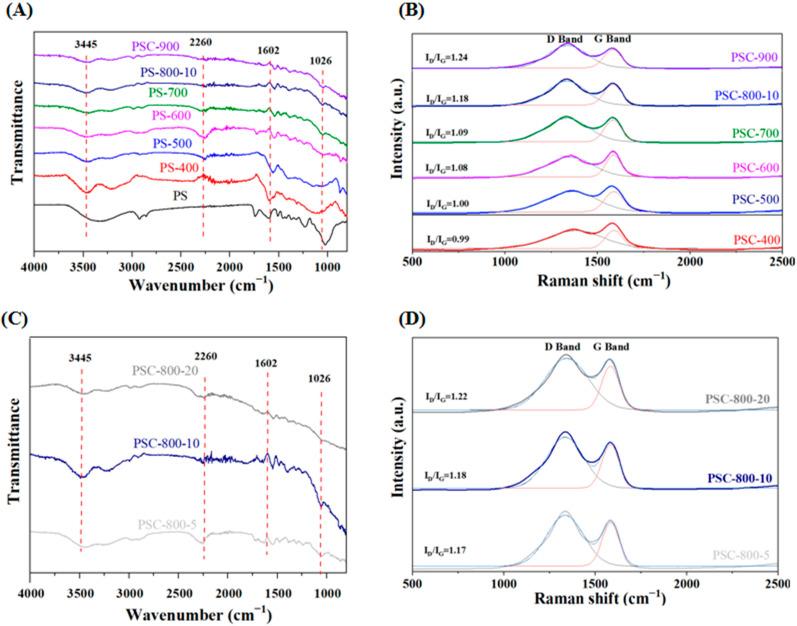
The FT-IR spectra and Raman spectra of the biochar derived from the pecan shell. (**A**,**B**) The biochar prepared at different temperatures and (**C**,**D**) the biochar prepared at different heating rates.

To explore the evolution of the functional groups on the PS and the biochar derived from the PS, FT-IR spectra were carried out and is displayed in Figure 3A,C. The wide characteristic peak at 3340 cm^−1^ was indexed to the stretching vibration of -OH. The peaks at 2926 cm^−1^ and 2946.12 cm^−1^ were attributed to the stretching vibrations of the sp^2^ C-H and sp^3^ C-H bonds, respectively. The three peaks at 1026 cm^−1^, 1236 cm^−1^, and 1602 cm^−1^ in the spectra of the PS, corresponded to the C-O-C stretching vibrational peaks present in the glycosidic, phenol, ether, and esters groups, the C-O stretching peak from the phenol, and the C=C stretching peak from the aromatic skeleton present in the lignin, respectively [36]. Notably, these peaks weakened in the biochar, which could be due to the degradation of the unstable components (such as hemicellulose, cellulose, and lignin) in the pecan shell during the carbonization process, thereby forming the new functional groups [37]. In addition, the intensity of the characteristic peaks was different, which indicated that the chemical properties of the functional groups on the surface biochar would be affected by the carbonization temperature [38]. The FT-IR spectra of the biochar prepared at 800 °C with different heating rates is shown in Figure 3C. It can be observed that the intensity of the peaks at 1026, 1602, 2260, and 3445 cm^−1^ weakened as the heating rate increased, which suggests that the functional groups would be affected by the heating rate. This result might have been caused by the degradation path and the rate of the components at the different heating rates.

The Raman spectra of the biochar prepared at different conditions are shown in Figure 3B,D. All the samples exhibited the two characteristic peaks at 1340 cm^−1^ and 1580 cm^−1^, originating from the disorder-induced D-band and the graphitic G-band, respectively, confirming the formation of the carbon structure from the pecan shell after the carbonization process. In detail, the former peak was attributed to the disorder structure or defects in the crystal lattice of the biochar, representing amorphous carbon, while the latter peak was related to the in-plane tangential stretching of the ordered sp^2^-bonded carbon, representing the graphite crystalline structure [39]. Furthermore, the graphitic degree of the biochar was identified by the value of I_D_/I_G_. This result displayed that the value of I_D_/I_G_ increased with the temperature increasing, which suggested that more defects formed in the biochar. This result would be owing to the formation of defects in the biochar structure caused by the release of CO_2_, CO, H_2,_ and CH_4_ derived from the decomposition of the cellulose, hemicellulose, and side chain groups of the lignin [40]. In addition, Figure 3D displayed the increase in the value of I_D_/I_G_ as the heating rate increased, manifesting more defects formed at the high heating rate, which would be due to the faster generation of gas at the carbonization process. Briefly, the degradation pathway and rate of hemicellulose, cellulose, and lignin in the pecan shell during the pyrolysis process would be affected by the pyrolysis conditions, which could change the physio-chemical properties on the surface of the biochar, further causing a different adsorption performance of the biochar prepared at different conditions.

**Figure 4:**
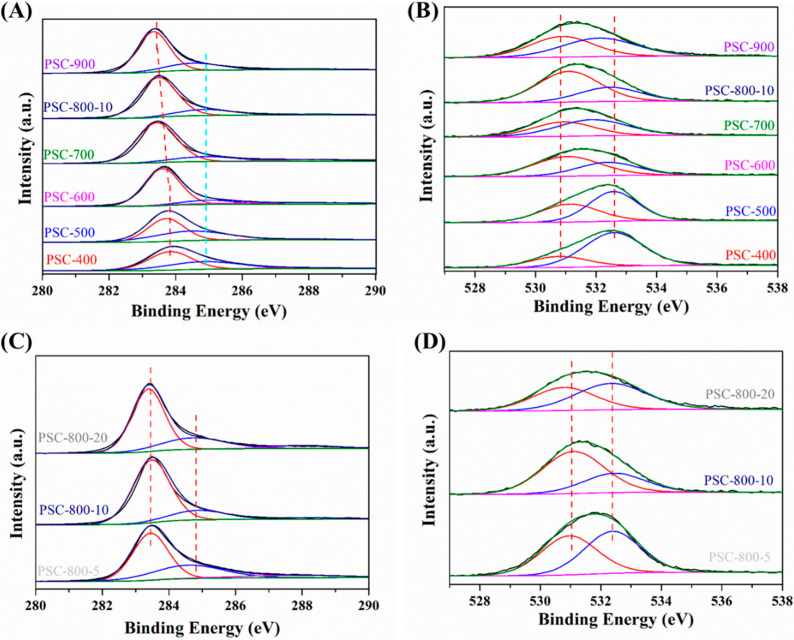
XPS analysis of biochar derived from pecan shell. (**A**,**B**) C 1s and O 1s spectra of biochar prepared at different temperatures and (**C**,**D**) C 1s and O 1s spectra of biochar prepared at different heating rates.

The element composition and functional groups on the surface of the biochar are important for the adsorption of pollutants. Herein, an XPS analysis was performed to identify the surface chemical properties of the biochar prepared at the different conditions, as presented in Figure 4 and Figure S3. The survey spectra of biochar are shown in Figure S3A, which displays that the biochar was mainly composed of carbon and oxygen. Also, Figure S3B shows that the content of carbon in the biochar was more than 85%, while, with the increase in the carbonization temperature, the content of the carbon increased, which could be due to the degradation of the oxygen-contained groups at the high temperature. Meanwhile, when the heating rate increased from 5 °C/min to 20 °C/min, the content of the carbon increased from 86.8% to 91.4%. This result indicates that the high heating rate was beneficial to enhancing the carbon content in the biochar, owing to the greater release of CO and CO_2_, which would optimize the pore structure [41].

The high-resolution C 1s spectra is shown in Figure 4A,C. The C 1s spectra could be deconvoluted into three peaks, which displayed a typical sp^2^ (C-C) at 283.5 eV along with a sp^3^ (C-C)/C=C at 284.8 eV [42]. In addition, a small peak at 288.0 eV corresponded to the O-C=O [33]. As we know, the sp^2^-hybridized carbon components were responsible for the impressive catalytic performances, due to the excellent electrophilicity of the active sites, which would be beneficial for adsorbing the pollutant [43]. In addition, the O-C=O groups could also adsorb the pollutant via electrostatic interaction [44]. Furthermore, the binding energy of the sp^2^-hybrizied C-C gradually decreased with the carbonization temperature, indicating the enhancement of the electron density, which may be attributed to the formation of defects in the biochar at the high temperature owing to the release of gaseous products (i.e., CO, CO_2_, H_2_ and CH_4_) [45]. Figure 4C shows the C 1s spectra of the biochar prepared with different heating rates, which shows that the surface properties of the biochar were not affected by the heating rate.

The oxygen-contained groups were important adsorption sites for the removal of pollutants [46]. Herein, the O 1s spectra were analyzed, as shown in Figure 4B,D. The O 1s peaks of all the samples were fitted to two parts: corresponding to O-C=O (531.0 eV) and corresponding to C-OH (532.4 eV) [47]. Figure 4B shows that the binding energy of O-C=O increased with the carbonization temperature, indicating that the electron transferred from the oxygen atoms to the carbon atoms, which could improve the electron deficiency of the oxygen-contained groups. This result may further the idea that the adsorption performance of biochar can be affected by the chemical state of oxygen-containing groups, thereby affecting the adsorption performance of the biochar. Moreover, for the biochar prepared at 800 °C with a heating rate of 20 °C/min, the binding energy of O-C=O shifted to a lower field, which may have affected the adsorption capacity of the biochar. Overall, the results of the XPS analysis verified that the carbonization temperature was an important influencing factor on the structure and surface chemical properties of the biochar, and that the heating rate during the carbonization process also could affect the electron distribution of functional groups.

### 2.2. The Adsorption of Congo Red (CR) with the Biochar Derived from Pecan Shells

#### 2.2.1. Initial Biochar Adsorbent Screening

**Figure 5:**
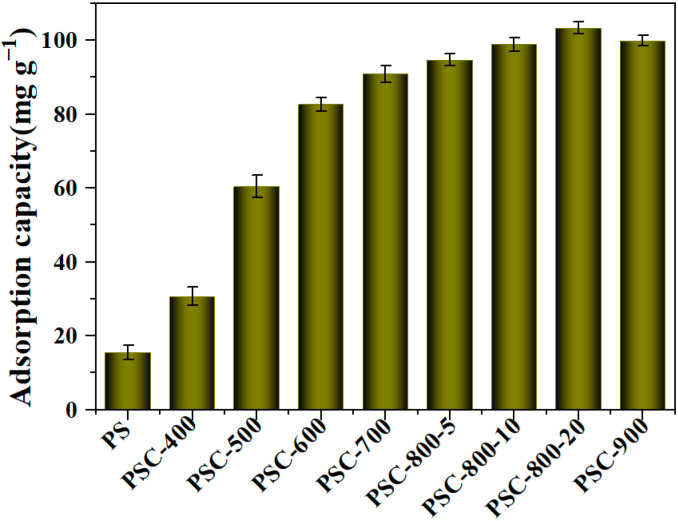
The adsorption capacity of biochar derived from pecan shell. (Adsorption conditions: T = 298 K; CR solution: 50 mL, 150 mg/L; adsorption time: 180 min; and dosage of biochar: 50 mg; pH = 7.2).

The adsorption performance of the biochar was investigated, as displayed in Figure 5. A pecan shell (PS) was applied to remove the CR dyes, which provided an adsorption capacity of 15.6 mg g^−1^. Then, the PS was carbonized to the biochar for the adsorption of CR. Figure 5 displays that the biochar exhibited more excellent adsorption performance than the PS precursor; specifically, the PSC-400 provided about two times greater adsorption capacity (30.7 mg g^−1^) in comparison to the PS. This result suggests that the adsorption performance enhanced after the carbonization treatment of the pecan shell. The experimental results showed that biochars prepared at a high temperature can perform higher adsorption capacity, which may be related to the specific surface area and functional groups on the surface of biochars. Notably, the PSC-800-20 exhibited the highest adsorption capacity, compared to other biochar. Therefore, the biochar PSC-800 was selected to investigate their adsorption behaviors during the adsorption process of CR.

#### 2.2.2. Adsorption Kinetics and Isotherms Studies of Congo Red

**Figure 6:**
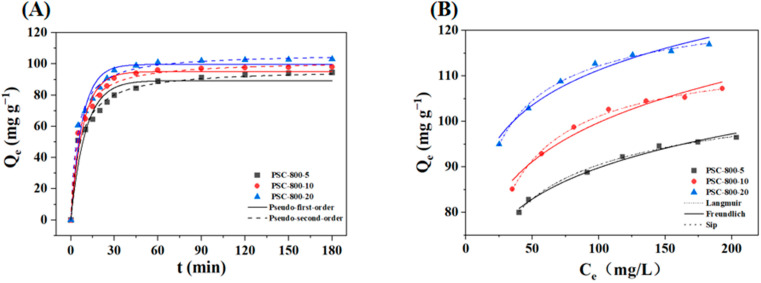
Adsorption kinetics (**A**) and isotherm (**B**), respectively, of CR on biochar (adsorption condition: pH = 7.2).

The adsorption kinetic curves and isothermal curves of PSC-800-5, PSC-800-10, and PSC-800-20 are exhibited in Figure 6. Figure 6A shows that the rapid adsorption stage of CR on the biochar occurred during the first 15 min, and then the adsorption rate gradually increased along with the adsorption time. When the adsorption time prolonged to 45 min, the adsorption capacity of the biochar reached 99.50 mg g^−1^, 116.37 mg g^−1^, and 130.48 mg g^−1^, corresponding to PSC-800-5, PSC-800-10, and PSC-800-20, respectively. This experimental result indicated that the biochar derived from the pecan shell was an efficient adsorbent for CR removal from dye wastewater. Also, the adsorption capacity of the biochar contributed to the specific surface area and adsorption sites, which could be tuned by the preparation condition (i.e., temperature and heating rate). Two kinds of adsorption kinetic models were applied to fit the CR adsorption process. The relative parameters are presented in Table 2. The results showed that the R^2^ of the pseudo-second-order model was higher than the R^2^ of the pseudo-first-order model. Hence, the pseudo-second-order model denoted a better fit to describe the adsorption behavior towards CR by the biochar, which revealed that the adsorption process was mainly dominated by chemisorption [48].

The three models (Langmuir, Freundlich, and Sip) were used to describe the adsorption behaviors of the PSC-800 biochar during the adsorption process (Figure 6B), where the Langmuir adsorption isotherm model assumed that the adsorption occurred on uniform adsorption sites and the Freundlich model assumed the adsorption occurred on the heterogeneous surface and unequal binding sits [49]. The experimental results showed that the adsorption capacity of all three biochars gradually increased with the increase in the initial CR concentration. As the reaction proceeded, more CR molecules were adsorbed on the biochar and the adsorption sites of the biochar continued to decrease, causing the adsorption capacity to reach a saturation state. In addition, Table 3 presented the relevant parameters calculated by the above-mentioned isotherm models. The R^2^ value (0.988–0.993) of the Langmuir model of the three catalysts were slightly above that of the Freundlich model, but both fit the experimental result well, indicating that the adsorption of the CR mainly occurred on the heterogeneous surface of the biochar [50]. Therefore, the Sip model was used to further explore the adsorption process, and it exhibited the best fitting trend (highest R^2^ value) for the three biochars compared to the Langmuir and Freundlich models. The γ values were close to 1 and determined whether the Sip model amounted to the Langmuir or the Freundlich model. The γ values of PSC-800-5, PSC-800-10, and PSC-800-20 were 0.505, 0.802, and 0.537, respectively, indicating that the surface of the PSC-800 biochar was mainly heterogeneous with varying distributions of active sites, but also including some homogeneous areas.

#### 2.2.3. Effect of the Solution pH

**Figure 7:**
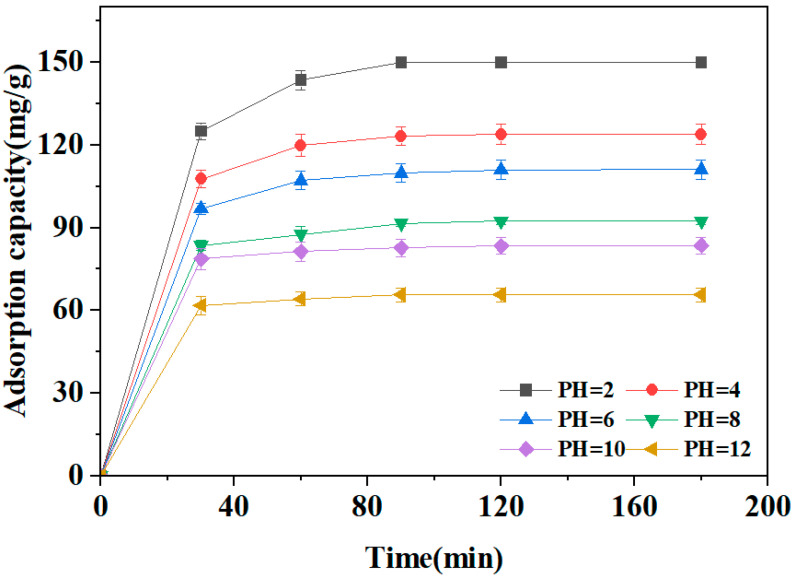
Effect of initial pH on the CR adsorption of the PSC-800-20 biochar. (Adsorption conditions: T = 298 K; CR solution: 50 mL, 150 mg/L; dosage of biochar: 50 mg).

The dye solution pH is an important effect factor during the adsorption process because it can affect the ionization of the adsorbate molecules and surface chemical properties of the adsorbent. Figure 7 illustrates the adsorption of CR on the PSC-800-20 biochar at different pH conditions. There was a sharp increase in the amount of adsorbed CR during the initial 30 min, after which it began to approach equilibrium. It can also be observed that stronger acidity resulted in a longer time to reach equilibrium. Notably, the biochar provided a higher maximum adsorption capacity when the value of the dye solution was 2, which indicated the more excellent adsorption performance of biochar at the dye solution with a lower pH value. The CR molecules could be protonated in acidic condition, which could weaken the repulsion between the CR molecules and the oxygen-containing functional groups on the carbon surface. Therefore, the biochar performed a higher adsorption capacity of CR at a lower pH solution.

#### 2.2.4. Reusability and Regeneration of Biochar Derived from Pecan Shell

**Figure 8:**
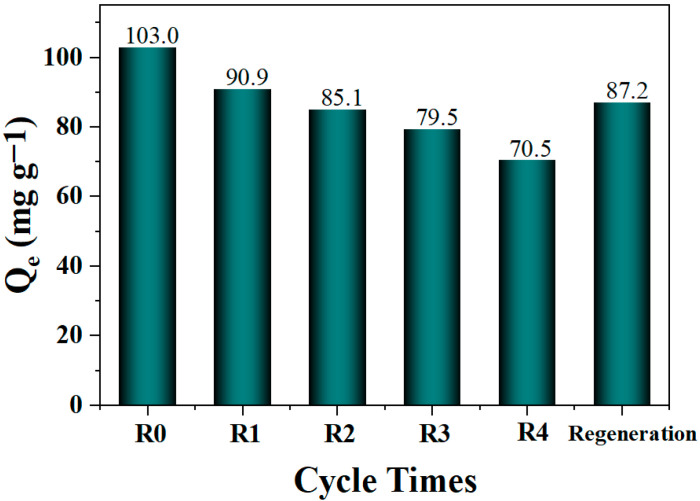
Reusability of PSC-800-20 biochar for the removal of CR. (Adsorption conditions: T = 298 K; CR solution: 50 mL, 150 mg/L; adsorption time: 180 min; and dosage of biochar: 50 mg, pH = 7.2).

The reusability of the biochar is an important factor, determining the economy during the application of adsorbing organic pollutant. Thus, the PSC-800-20 was evaluated by performing consecutive runs using the recovered biochar. Specifically, the PSC-800-20 biochar was separated via centrifugation after the adsorption of the CR dye, then directly subjected to the next run. Figure 8 shows that the adsorption capacity of the biochar gradually decreased as the experiment continued, which may be due to the surface adsorption sites occupied by the CR molecules. Then, the recovered PSC-800-20 biochar was regenerated at 500 °C under a N_2_ atmosphere. The regenerated PSC-800-20 provided 87.2 mg g^−1^ of CR adsorption capacity. The slight decrease in the adsorption capacity may have been indexed to the residual CR molecules blocking the pore or the collapse of the pore part during the regeneration process.

#### 2.2.5. Possible Adsorption Mechanisms of CR on PSC-800-20 Biochar

**Figure 9:**
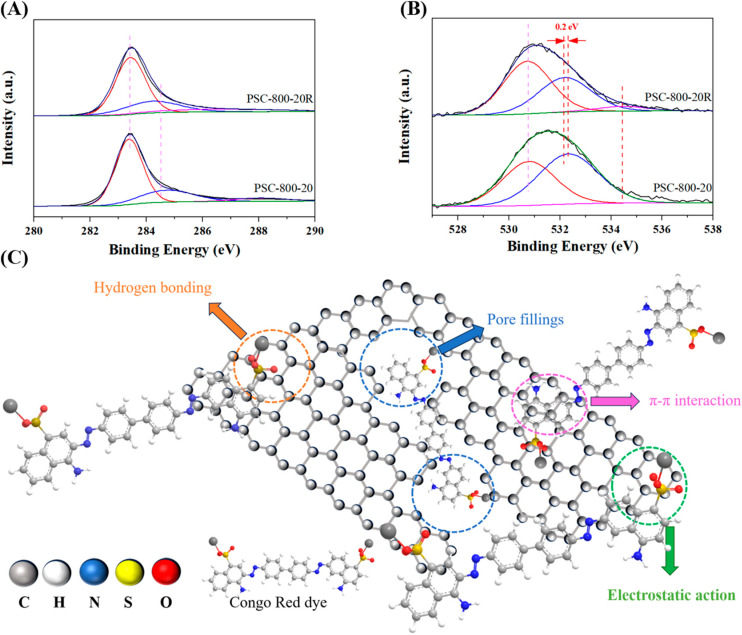
XPS spectra of the fresh and recovered biochar (**A**,**B**) and the possible mechanism of the adsorption process (**C**).

Based on the previous results of the characterizations and experiments, a possible adsorption mechanism was proposed, as shown in Figure 9. A combination of the analysis of the pore structure and the adsorption performance of the biochar with the higher specific surface area could provide more excellent adsorption performance. This indicated that the pore structure of the biochar performed an important role during the adsorption process. In addition, the XPS analysis of the recovered PSC-800-20 biochar was explored. The results showed that the binding energy of C 1s at 284.8 eV shifted significantly, which may have been caused by the disturbance of the benzene ring structure in the adsorbed CR molecules (Figure 9A). Meanwhile, the binding energy of the O 1s had apparently changed. The binding energy at 532.4 eV (C-OH group) shifted to a low field (0.2 eV), indicating the increase in the electron density around the oxygen atom in the C-OH group (Figure 9B). Hence, a possible adsorption mechanism was proposed, as shown in Figure 9C. The CR molecules would fill the pores of the biochar because of the porous structure of the biochar. In addition, the abundant hydroxyl on the surface of the biochar could interact with the CR molecules via the hydrogen bonding and electrostatic interaction. Meanwhile, the benzene ring could interact with the biochar structure via π-π stacking.

## 3. Experimental Section

### 3.1. Preparation of the Biochar Derived from the Pecan Shell

The pecan shell (PS) was obtained from Linan, Hangzhou, China. Before the preparation process, the sample was milled into 120 meshes by a grinder (ML-1000, Wuyi Haina Instrument Technology Co., Ltd., Wuyi, China) and washed five times with deionized water to remove the impurities on the surface of the PS, then the PS powder was dried at 105 °C overnight.

The biochar derived from the PS was prepared at the temperature range of 400 to 900 °C. In brief, the pecan shell powder (10 g) also was added into a crucible and then put in a tube furnace under the N_2_ atmosphere (100 mL/min), heated to 800 °C at 10 °C/min, and kept for another 2 h. In addition, the biochar was also prepared at 800 °C with different heating rates (5 °C/min, 10 °C/min and 20 °C/min). Finally, the biochar was collected after the following steps, and named as PSC-X-Y, where X was the temperature and Y was the heating rate.

This study investigated the effects of pyrolysis temperature, heating rate, pH, reusability, and regeneration. The adsorption test was performed in a conical flask with 100 mL of Congo Red (CR) and 50 mg biochar at a 150 mg/L concentration. The samples were placed in an incubator at 25 °C, 150 rpm for 3h for adsorption. After that, the water sample was filtered with 0.45 µm filter membranes. The antibiotic content was measured via ultraviolet spectrophotometry (TU-1901, Beijing Purkinje General Instrument Co., Ltd., Beijing, China) with Congo Red (CR) of 488 nm, and the biochar adsorption capacity was calculated. Each set of trials was repeated three times to ensure accuracy, and the recovered biochar after five runs had been regenerated in a tube furnace at 500 °C under a N_2_ atmosphere for 30 min, and then the powder was collected when the temperature cooled down to room temperature.

The adsorption kinetics of Congo Red were carried out using PSC-800-5, PSC-800-10, and PSC-800-20. The adsorption conditions were consistent with the above adsorption test conditions. The treated liquid was collected at 5, 10, 15, 20, 25, 30, 45, 60, 90, 120, 150, and 180 min during adsorption. The Congo Red content in the solution was measured using ultraviolet spectrophotometry. Each experimental group consisted of three replicates. The experimental data were matched by the pseudo-first-order and pseudo-second-order models. In addition, the CR adsorption behavior of the biochar was analyzed and explained. In the adsorption isotherm fitting test, the initial CR solution concentrations were 120, 150, 180, 210, 240, 270, and 300 mg/L, with an adsorption time of 3 h. The Langmuir and Freundlich models fit the adsorption isotherms to the experimental data. In addition, the reusability of the biochar was evaluated for 5 consecutive runs. The used biochar was collected via filtration, then directly used for the next run.

### 3.2. Characterization of the Biochar Derived from the Pecan Shell

The microstructure of the biochar was characterized by the scanning electron microscope (SEM, SU-8010, Hitachi, Tokyo, Japan). The crystalline structure of the biochar was analyzed on an X-ray diffractometer (XRD 6000, Shimadzu, Tokyo, Japan) with Cu Kα radiation. The Brunner–Emmett–Teller (BET) surface area and porosity of the biochar were recorded on an Automatic Surface Area and Pore Analyzer (ASAP2460, Micromeritics, Norcross, GA, USA). The Fourier transform infrared (FT-IR) biochar was analyzed by a BRUKER VERTEX 80 V spectrometer (BRUKER, Bremen, Germany). The Raman spectra of the biochar were recorded on a Renishaw InVia micro-Raman spectrometer with laser excitation at 633 nm. X-ray photoelectron spectrometry (XPS, Thermo Fisher Scientific, Waltham, MA, USA) was applied to analyze the surface chemical states of the biochar.

S_BET_, V_micro_, and V_meso_ represented the specific surface area, the micropore volume, and the mesopore volume, which were determined by the Brunner–Emmett–Teller (BET) method, the t-plot method, and the Barrett–Joyner–Halenda (BJH) method, respectively.

## 4. Conclusions

In summary, a low-cost and environmentally friendly biochar was prepared from the agricultural waste of a pecan shell and applied to remove the CR dye from wastewater. The results of the characterization proved that the preparation conditions (temperature and heating rate) could significantly affect the structure and surface properties of biochars, which further affected the adsorption performance. The biochar prepared at a relatively higher temperature (800 °C) and heating rate (20 °C/min) presented a higher specific surface area and adsorption capacity of CR, which would be attributed to the excellent pore structure and oxygen-containing groups on the surface of biochars. Furthermore, the adsorption process was investigated, and the results showed that the pseudo-second-order kinetic model and the Langmuir model exhibited better fits for describing the adsorption process of the biochar derived from the pecan shell. This study provides a novel strategy for utilizing agricultural waste resources in the purification of dye wastewater. Nevertheless, some approaches should be further developed for improving their specific surface area and tuning the functional groups on the surface of biochars to enhance the adsorption performance of the biochars derived from agricultural waste. In addition, the applicability of dye mixtures in actual wastewater treatment should be further explored.

## Figures and Tables

**Table 1:** BET surface area and pore properties of the biochar.

Samples	S_BET_ (m^2^ g^−1^)	V_total_ (cm^3^ g^−1^)	V_micro_ (cm^3^ g^−1^)	V_meso_ (cm^3^ g^−1^)
PSC-400	3	0.005	0	0.004
PSC-500	11	0.009	0.004	0.005
PSC-600	368	0.144	0.135	0.009
PSC-700	398	0.145	0.142	0.004
PSC-800-5	93	0.043	0.030	0.012
PSC-800-10	436	0.158	0.147	0.011
PSC-800-20	450	0.173	0.139	0.034
PSC-900	96	0.406	0.034	0.371

**Table 2:** Adsorption kinetic parameters for CR on different biochar.

Samples	Pseudo-First-Order Model		Pseudo-Second-Order Model	
	K_1_ (min^−1^)	R^2^	K_2_ (min^−1^)	R^2^
PSC-800-5	0.098	0.936	0.002	0.986
PSC-800-10	0.118	0.963	0.002	0.991
PSC-800-20	0.126	0.960	0.002	0.992

**Table 3:** Parameters derived from isotherms of CR on different biochar.

Samples	Langmuir Isotherm Model				Freundlich Isotherm Model			Sip Model			
	q_max_ (mg g^−1^)	K_L_ (min^−1^)	R_L_	R^2^	K_F_ (L mg^−1^)	1/n	R^2^	q_max_ (mg/g)	k (mg/(g⋅min))	γ	R^2^
PSC-800-5	99.50	0.011	0.377	0.988	69.09	0.104	0.967	115.66	0.129	0.505	0.991
PSC-800-10	116.37	0.130	0.048	0.993	54.78	0.130	0.957	119.82	0.092	0.802	0.993
PSC-800-20	130.48	0.378	0.017	0.993	52.93	0.114	0.985	132.48	0.200	0.537	0.994

## Data Availability

The datasets used and/or analyzed during the current study are available from the corresponding author on reasonable request.

## Supplementary Materials

The following supporting information can be downloaded at https://www.mdpi.com/article/10.3390/molecules29235532/s1. Figure S1: The EDS analysis of the PSC-800-10 biochar. Figure S2: The N2 adsorption–desorption isotherms (A,B), and the pore size distribution curves (C,D) of the biochar prepared at a different temperature and heating rate. Figure S3: X-ray photoelectron spectroscopy analysis of the obtained samples. (A) Survey spectra of the biochar prepared at different conditions. (B) The carbon content of the obtained biochars from XPS analysis. Table S1: the contents of carbon and nitrogen element of biochar.
