# Supplementary material for: The Biochar Derived from Pecan Shells for the Removal of Congo Red: The Effects of Temperature and Heating Rate
Source: Molecules. 2024 Nov 22;29(23):5532. doi: 10.3390/molecules29235532 (PMC11643957; doi:10.3390/molecules29235532)
Supplement: Supplementary file 1 [file molecules-29-05532-s001.zip › molecules-3271763-supplementary.pdf]

Supporting Information

The Biochar Derived from Pecan Shells for the Removal of Congo Red: The Effects of Temperature and Heating Rate

Wanqiang Xu [1], Bo Cai [2,*], Xujie Zhang [1], Yating Zhang [1], Yongjian Zhang [1,3,*] and Hehuan Peng [1,3,*]

[1] College of Optical, Mechanical and Electrical Engineering, Zhejiang A & F University, Hangzhou 311300, China
[2] College of Chemistry and Materials Engineering, Zhejiang A & F University, Hangzhou 311300, China
[3] Key Laboratory of Agricultural Equipment for Hilly and Mountainous Areas in Southeastern China (Co-Construction by Ministry and Province), Ministry of Agriculture and Rural Affairs, Hangzhou 311300, China
* Correspondence: caibo@zafu.edu.cn (B.C.); zhangyongjian@zafu.edu.cn (Y.Z.); penghh@zafu.edu.cn (H.P.)

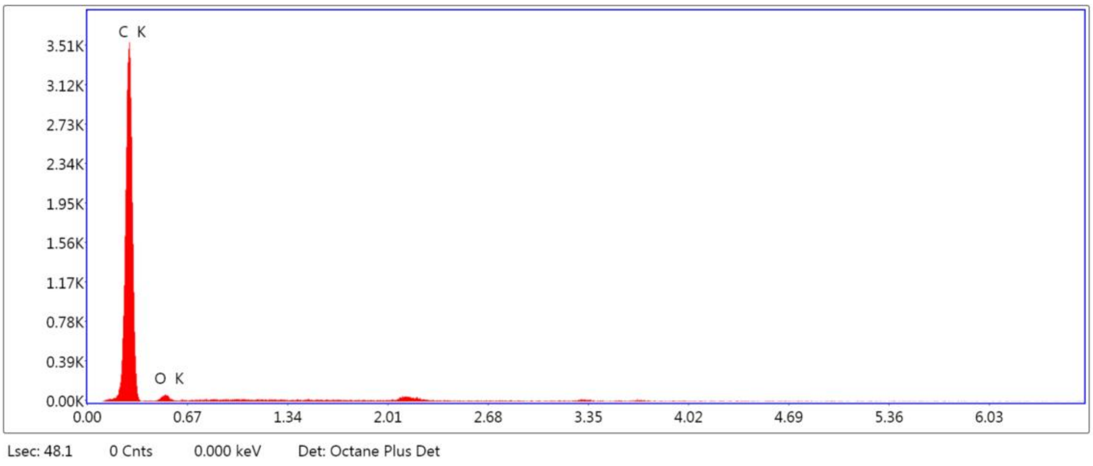

Figure S1 The EDS analysis of the PSC-800-10 biochar.

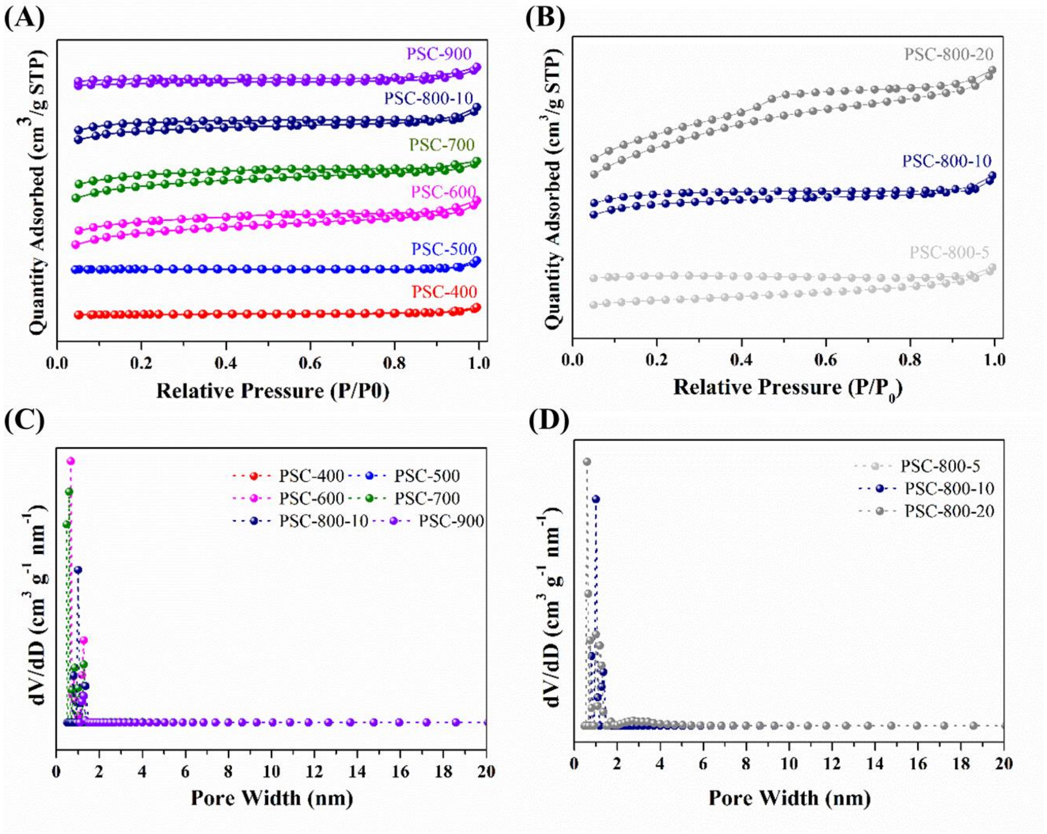

Figure S2 the N_2 adsorption–desorption isotherms (A) (B), and the pore size distribution curves (C) (D) of the biochar prepared at a different temperature and heating rate.

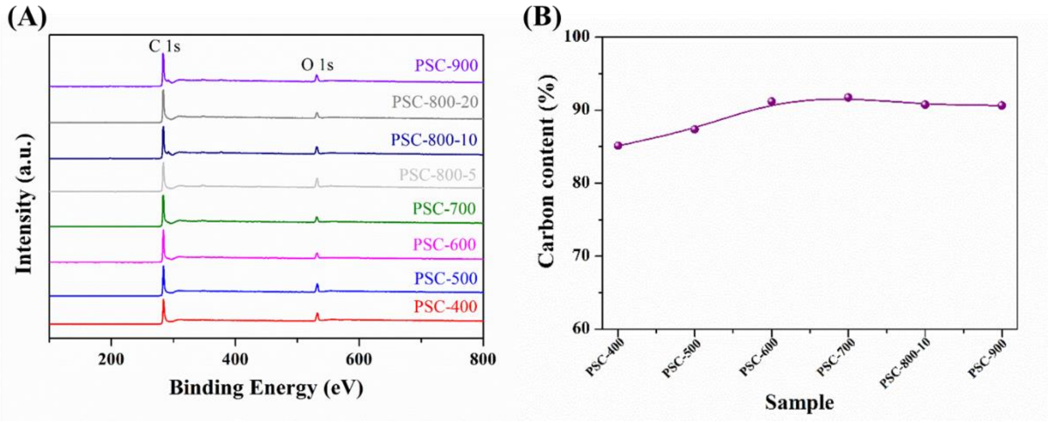

Figure S3 X-ray photoelectron spectroscopy analysis of the obtained samples. A) Survey spectra of the biochar prepared at different conditions. B) The carbon content of the obtained biochars from XPS analysis.

Table S1. the contents of carbon and nitrogen element of biochar

**Element**	**Weight %**	**Atomic %**	**Error %**
C K	94.92	96.14	2.57
O K	5.08	3.86	15.41
